# Application of Native or Exotic Arbuscular Mycorrhizal Fungi Complexes and Monospecific Isolates from Saline Semi-Arid Mediterranean Ecosystems Improved *Phoenix dactylifera*’s Growth and Mitigated Salt Stress Negative Effects

**DOI:** 10.3390/plants10112501

**Published:** 2021-11-18

**Authors:** Elmostapha Outamamat, Mohammed Bourhia, Hanane Dounas, Ahmad Mohammad Salamatullah, Abdulhakeem Alzahrani, Heba Khalil Alyahya, Nawal A. Albadr, Mohamed Najib Al Feddy, Bacem Mnasri, Lahcen Ouahmane

**Affiliations:** 1 Labeled Research Unit-CNRST N°4, Laboratory of Microbial Biotechnology, Agro-Sciences and Environment (BioMAgE), Cadi Ayyad University, Marrakesh 40000, Morocco; outaelmostaphamamat@gmail.com (E.O.); bourhiamohammed@gmail.com (M.B.); hadounas@gmail.com (H.D.); 2 Department of Food Science & Nutrition, College of Food and Agricultural Sciences, King Saud University, P.O. Box 2460, Riyadh 11451, Saudi Arabia; asalamh@ksu.edu.sa (A.M.S.); aabdulhakeem@ksu.edu.sa (A.A.); hkalyahya@ksu.edu.sa (H.K.A.); nalbader@ksu.edu.sa (N.A.A.); 3 Plant Protection Unit, Laboratory of Phyto-Bacteriology, National Institute of Agronomic Research, Marrakesh 40000, Morocco; alfeddynajib@yahoo.fr; 4 Centre of Biotechnology of Borj-Cédria, Hammam-Lif 2050, Tunisia; mnbacemm@yahoo.com

**Keywords:** mycorrhizal fungi, single-species, biofertilizers, date palm, salt stress

## Abstract

The date, the palm tree (*Phoenix dactylifera* L.) is an important component of arid and semi-arid Mediterranean ecosystems, particularly in Morocco where it plays a considerable socio-economic and ecological role. This species is largely affected by desertification, global warming, and anthropic pressure. Salinity is a very worrying problem that negatively affects the growth and the physiological and biochemical activities of the date palm. In these arid zones, the main challenge is to develop new environmentally friendly technologies that improve crop tolerance to abiotic restraints including salinity. In this sense, Arbuscular mycorrhizal fungi (AMF) have received much attention due to their capability in promoting plant growth and tolerance to abiotic and biotic stresses. It is thus fitting that the current research work was undertaken to evaluate and compare the effects of native AMF on the development of the growth and tolerance of date palm to salt stress along with testing their role as biofertilizers. To achieve this goal, two complexes and two monospecific isolates of native and non-native AMF were used to inoculate date palm seedlings under saline stress (0 g·L^−1^ Na Cl, 10 g·L^−1^, and 20 g·L^−1^ Na Cl). The obtained results showed that salinity drastically affected the physiological parameters and growth of date palm seedlings, whilst the application of selected AMF significantly improved growth parameters and promoted the activities of antioxidant enzymes as a protective strategy. Inoculation with non-native AMF complex and monospecific isolates showed higher responses for all analyzed parameters when compared with the native complex and isolate. It therefore becomes necessary to glamorize the fungal communities associated with date palm for their use in the inoculation of *Phoenix dactylifera* L. seedlings.

## 1. Introduction

Climate change has disrupted the environmental balance, causing significant salinization of soils. Saline soils occupy about 8% of the planet’s surface with an increasing trend [1]. Soil salinity is a growing problem affecting agricultural land with serious consequences on the plant growth and development, resulting in a reduction of more than 20% of agricultural yield [2,3]. The rise in soil salinity causes osmotic and specific ionic effects, which in turn result in secondary stress in plants, namely, oxidative stress [4,5].

Date palm (*Phoenix dactylifera* L.) is currently the largest cultivated plant fruit in arid lands of North Africa and the Middle East thanks to its socio-economic and environmental values [6,7,8,9]. In Morocco, a large land area dedicated to date palm trees’ cultivation has suffered from intense degradation under the effect of various abiotic and biotic stresses, including salinity [10]. The latter is one of the most harmful issues, which limits the growth and the development of plants [11].

Previous studies investigated the responses of the date palm to salt stress [11,12] and reported that salt exerts deleterious effects on both biochemical and physiological activities including seed germination, photosynthesis, mineral homeostasis, osmotic equilibrium, as well as respiratory processes [13]. Salinity is the main responsible factor for oxidative stress that is associated with the release of reactive oxygen species (ROS), which in turn negatively affect plant metabolism and growth [11,12,14]. Plants can eliminate these toxic forms by different defense mechanisms such as the antioxidant system modulated by diverse enzymes including peroxidase, catalase (CAT), superoxide dismutase (SOD), as well as polyphenol oxidase (PPO) [15].

In addition, former studies have already investigated alternative solutions that could enhance the defense mechanisms in saline environments. In this sense, it was reported that the arbuscular mycorrhizal fungi (AMF) inoculates played a role in promoting plant growth and mineral nutrition along with reducing the adverse effects of salt stress [16,17]. These beneficial soil microorganisms showed an ability to overcome stressful environments [13,17,18]. AMF belong to the phylum *Glomeromycota* known by mutualistic symbiosis with roots of more than 80% of plants [19]. Telluric fungi have been reported to ameliorate plant growth by increasing their water and mineral nutrition, particularly phosphorus uptake from non-labile sources of mycorrhizal plants. In addition, water and mineral uptakes from the soil solution lead to higher photosynthetic activity of mycorrhizal plants. In adverse environmental conditions, the AMF contributed to the increasing tolerance to abiotic and biotic stresses by their osmotic regulation and alleviation of oxidative stress [17,18,20]. Mechanisms of mycorrhizal plant tolerance to abiotic stresses, for instance, salinity, drought, as well as pollution, have critically been revised elsewhere [13,21,22,23]. Despite the ubiquity of AMF in different harsh environmental conditions and their promising findings in protecting plants from stresses, few works have been undertaken to investigate the potential effects of AMF in fighting against salt stress in date palm [11,24,25]. It is thus fitting that the use of AMF as bio-fertilizers in the technical itinerary is proposed as an ecological technique producing high-quality seedlings able to tolerate salt stress and face the consequences of global warming.

The present study was carried out to evaluate and compare the effects of AMF isolated from highly saline regions in the improvement of date palm seedlings’ tolerance to salt stress. In the present work, two complexes and two monospecific isolates of AMF native and non-native to the date palm groves were used in the experiment. Seedlings were grown under unstressed (0 g·L^−1^ NaCl) or stressed conditions (10.00 g·L^−1^ and 20.00 g·L^−1^ NaCl) for testing purposes.

## 2. Material and Methods

### 2.1. Mycorrhizal Fungi Propagules

The sampling was conducted from two areas located in the regions of Zagora (degraded palm grove) and Marrakesh (saline source) in Morocco. The climate is arid to semi-arid. Soil samples were collected from about the trunk of *Phoenix dactylifera* at depths ranging from 10 to 30 cm and stored at 4 °C until further use (Table 1).

### 2.2. Preparation of the Mycorrhizal Inoculum

The first preparation step was the establishment of the mycorrhizal trap culture using Maize (*Zea mays* L.) as the necessary endophyte plant. Corn seeds were disinfected before being germinated in pots containing soils collected from Zagora (complex A10) and Marrakesh (complex A9) (Table 1). After three months of cultivation, AMF spores were meticulously extracted by using the wet sieving method [26]. Next, the extracted spores were disinfected with a chloramine T solution (0.2 g L^−1^) and streptomycin (0.2 g L^−1^) [27]. The second step was to germinate disinfected corn seeds and drive seedlings into an autoclaved sandy soil substrate (140 °C for 3 h). Afterward, maize seedlings were inoculated with a suspension of sterilized mycorrhizal spore mixture obtained from the three month trap culture. Next, roots colonized with the mycorrhizal fungal complex were accurately rinsed three times with distilled water before being cut into 1 cm fragments and used as fresh mycorrhizal inoculum [28,29]. The obtained inoculants were given numbers such as complexes A9 and A10 (Table 1).

### 2.3. Single-Species Cultures and Microscopy Identification

The selection process of AMF was based on the most abundant morphotypes among the spore community that form the complexes A9 and A10. The production of monospecific isolates from single spore suspension was effectuated using the technique of micropipette tips described elsewhere [30,31]. Briefly, monosporal culture was selected from healthy and infectious spores previously originated from rhizospheric soil. Next, the single-species culture was isolated from AMF spores before being set up using pipette tip microcosms to promote mycorrhizal formation. After 2 months, the bottom of the pipette tip was cut to enable the seedlings to expand after being transferred to a bigger soil volume. Transferring these tip pipettes into a bigger soil volume (100 mL) permitted the multiplication of fungal spores. Additionally, more seeds of endophytic plants were added to the soil. After about 3 months, the soil was dried and all pots were screened to check whether the mycorrhizal establishment successfully met the regular rate of about 10%–15%. The soil of positive samples was used as inocula to set up bigger pots for spore multiplication (1–2 L). Finally, refined monospecific isolates were successfully produced (A9RF and A10RF).

### 2.4. Inoculation and Experimental Design

The experimental design consisted of evaluating and comparing the effects of native (Zagora palm grove) and non-native AMF (isolated from a saline date palm rhizosphere in the Marrakesh region) in the improvement of the tolerance of date palm to salt stress and their role as biofertilizers. To achieve this goal, two complexes (A9 and A10) and their related fungal isolates (A9RF and A10RF) were used in the experiment. Seedlings were grown under unstressed (0 g·L^−1^ NaCl) or stressed (10 and 20 g·L^−1^ NaCl) conditions. Inoculation of germinated seeds was performed by mixing an individual germinated seed with 5 g of fresh mycorrhizal root fragments in a hole in the middle of a pot containing 2.00 kg of sterilized soil collected under the date palm trees. The experiment was cautiously run under greenhouse conditions at Cadi Ayyad University in Marrakesh. The average temperature (day/night) was about 36/25 °C and the relative humidity was 55%/86%, whilst the photoperiod was about 16 h of light/8 h of darkness. After eight months of cultivation under daily irrigation until saturation, the date palm seedlings were subjected to salt stress (0 g·L^−1^, 10 g·L^−1^, and 20 g·L^−1^ NaCl) for two months (March and April 2021).

### 2.5. Mycorrhizal Traits, Growth, Mineral Nutrition, Physiological and Biochemical Parameters

#### 2.5.1. Root Mycorrhizal Colonization

A small fragment of roots (lateral root system) was accurately washed with distilled water and cleared with 10% KOH (Sigma-Aldrich, Schnelldorf, Germany) at 90 °C for 30 min then acidified with HCl (1%) for 10 min and stained in Trypan blue at 90 °C for a further 20 min [32]. The frequency and intensity of AMF were evaluated using the method elaborated by Trouvelot [33].

#### 2.5.2. Growth Parameters

After two months under salt stress, the ten-month aged date palm seedlings were harvested and subjected to measurements. The height of the shoots (cm) was measured. The shoots and roots fresh and dry weights (g) were determined after 24 h at 105 °C.

#### 2.5.3. Mineral Contents Analysis

Previously dried shoots and roots (62 °C for one week in the oven) were ground into a fine powder before being digested using 30% H_2_O_2_ and 98% H_2_SO_4_. Next, the total nitrogen Kjeldhal (TNK) was measured using the Kjeldhal method, whilst the phosphorus content was quantified according to the Olsen method [34]. The content of Ca^2+^, K^+^, Na^+^, and Mg in seedlings tissues were measured using Inductively Coupled Plasma Optical Emission Spectrometry (ICP OES Thermo IRIS Interpid II XDL Duo, Waltham, MA, USA).

#### 2.5.4. Chlorophyll Contents

Fifty milligrams of fresh leaf material was macerated with 3 mL of 90% acetone solution before being centrifuged at 100 rpm for 10 min. Afterward, the sample was incubated for 3 h in the darkness to ready it for optical density (OD) measuring at 663 and 645 nm using a S-22UV/Vis. spectrophotometer (Beoco, Germany). Chlorophylls, such as a, b, and total chlorophyll contents, were measured according to previously reported protocols [35].

Chlorophyll a (µg·mL^−1^) = (11.93 × DO664) − (1.93 × DO647) (1)

Chlorophyll b (µg·mL^−1^) = (20.36 × DO647) − (5.5 × DO664) (2)

#### 2.5.5. Protein Contents

One hundred milligrams of leaf samples were homogenized with 0.10 mL of 50.00 mM potassium phosphate buffer (7.5 pH), and 0.1 mM EDTA, and 1% pvpp (polyvinylpolypyrrolidone) to obtain the protein extract. Next, the mixture was centrifuged at 4 °C for 20 min (12,500× *g*) to obtain supernatant, which in turn was used in determining protein contents along with the enzymatic activity. In this sense, the total protein was determined according to Bradford [36]. Briefly, 100.00 µL of the protein extract was mixed with 100.00 µL of diH_2_O before being added to 2 mL of Bradford’s reagent. Thereafter, the sample was incubated for 5 min to ready it for measuring optical density (OD) at 595 nm. The protein content was estimated using a serum bovine albumin standard curve as reported elsewhere [36].

#### 2.5.6. Malondialdehyde Contents

The content of Malondialdehyde in the roots and leaves of the studied plant was determined according to Dhindsa et al. (1981) [37]. Briefly, 0.25 g of plant material was mixed with 10 mL of 0.10% TCA before being centrifuged at 18,000× *g* for 10 min. Next, a 2 mL aliquot of the supernatant was added to 2 mL of 20% TCA with 0.5% TBA. Afterward, the obtained mixture was accurately heated at 100 °C for 30 min and quickly cooled to prepare it for centrifugation at 10,000× *g* for 10 min for clarification purposes. Finally, the absorbance of the supernatant was measured at 532 nm and the nonspecific turbidity was cautiously adjusted by A600 subtracting from A532 [37].

#### 2.5.7. Antioxidant Enzyme Assay

In the current research work, enzyme assay was studied according to optimized protocols reported in earlier work [23]. To achieve this goal, 0.5 g of fresh plant leaves were carefully frozen in liquid nitrogen before being cut and maintained at 4 °C in 5 mL solution constituted of 0.1 g polyvinylpolypyrrolidone (PVPP), 0.1M potassium phosphate buffer (pH 7.0) as well as 0.1 mmol ethylenediaminetetraacetic acids (EDTA) (Sigma-Aldrich Schnelldorf, Germany). Thereafter, the mixture was centrifuged at 18,000× *g* and 4 °C for 10 min to obtain the supernatant, which was kept at −20 °C for biochemical analysis. The activity of total SOD was dosed using the method described by Beyer and Fridovich (1987) [38] based on the evaluation of enzyme ability to inhibit the photochemical decrease of nitro blue tetrazolium (NBT) [38]. In the present work, the required amount of enzyme to impede the reduction of NBT by 50% at 25 °C was defined as one unit of SOD. The SOD activity was expressed in unit min^–1^ mg^–1^ protein. The CAT activity (CAT, EC 1.11.1.6) was defined as a reduction in the absorbance (240 nm) for 3 min following H_2_O_2_ decomposition [39]. The reaction mixture consisted of 0.10 mM EDTA, 0.10M potassium phosphate buffer (pH 7.0), 20.00 mM H_2_O_2_ and 100 μL of enzyme extract in a 2 mL volume. The activity of (PPO) was measured using the method described by Moore and Flurkey [40]. The assay reagent possessed 20 mM catechol in 0.10 M phosphate buffer (pH 7). The reaction was launched by addicting 100 μL of enzymatic extract. The activity of PPO was expressed in enzyme unit mg^−1^ per protein. The activity was evaluated as the rise in the absorbance at 420 nm. Control bioassays were carried out without substrate to assess the auto-oxidation of the substrates. The antioxidant effects were expressed in unit mg protein^−1^ min^−1^ as reported elsewhere [40].

### 2.6. Statistical Analysis

The statistical analysis was effectuated using two-way ANOVA with the XL stat software. Mycorrhizal inoculation was the first factor and salinity level was the second one. The significance of the differences between treatments and factor interactions were calculated at 5% and mean comparisons were defined using Tukey’s HSD test (*p* ≤ 0.05). The ANOVA was completed with Principal Component Analysis (PCA) to separate the different treatments and determine the groups according to the measured parameters.

## 3. Results

### 3.1. Effects of Salt Stress on Mycorrhizal Infection of Date Palm Seedlings

**Figure 1:**
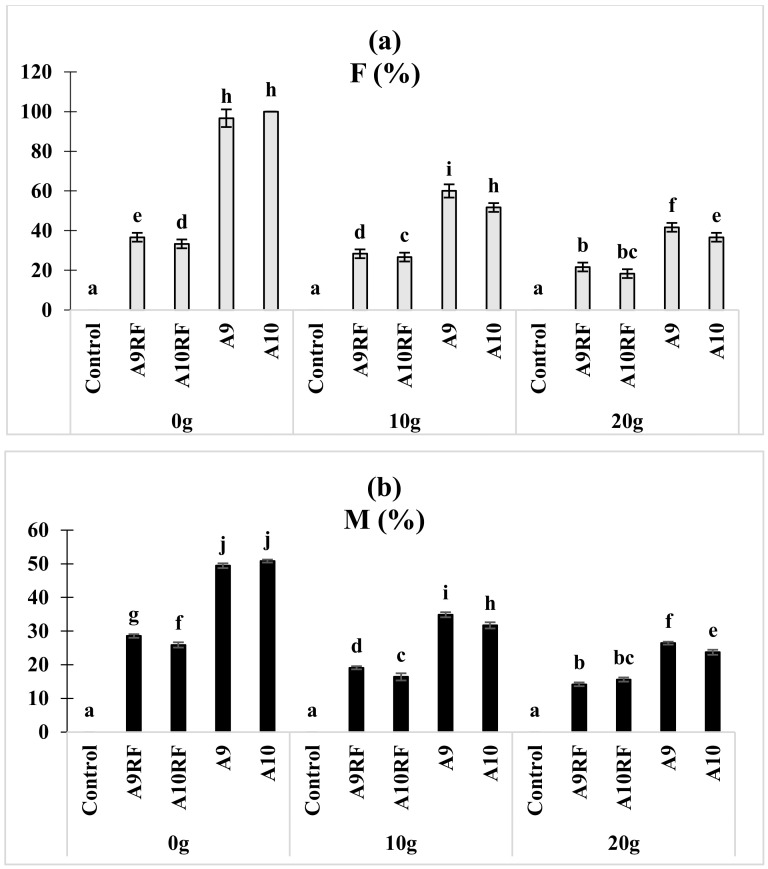
Effect of salt stress on roots’ infection rate in ten-month aged date palm seedlings after inoculation with different fungal complexes and isolates. Mycorrhizal frequency (F %) (**a**) and mycorrhizal colonization (M %) (**b**). Graphs indexed by the same letter are not significantly different according to Tukey (HSD) test (*p* < 0.05).

The microscopic analysis of the mycorrhizal status of date palm seedlings revealed the presence of all the expected mycorrhizal fungi structures (hyphae, vesicles, arbuscules, and spores), in seedlings roots after eight months of cultivation followed by two months under salt stress. The roots were infected by the mycorrhizal complexes before being isolated with frequency varying from 33% to 100% for the non-stressed seedling. The mycorrhizal frequency varied between 26% and 60% for seedlings under 10 g·L^−1^ and between 18% and 41% for those under 20 g·L^−1^ (Figure 1a). The analysis of the mycorrhizal colonization of date palm seedlings showed that this parameter ranged between 25% and 50% for non-stressed seedlings and between 16% and 34% for inoculated seedlings under 10 g·L^−1^ salt and finally between 15% and 26% for inoculated seedlings under 20 g·L^−1^ salt (Figure 1b). The successful establishment of mycorrhizal symbiosis was recorded for the complex A9 even in the case of extreme stress at 20 g·L^−1^. Analysis of the mycorrhizal frequencies and the colonization rates of the date palm roots by the different fungi inoculated to the seedlings showed that the complexes largely dominated the respective monospecific isolates. The mixture A9 recorded the best mycorrhizal performances.

### 3.2. Effects of Different Inocula on the Growth of Date Palm Seedling under Salt Stress

**Figure 2:**
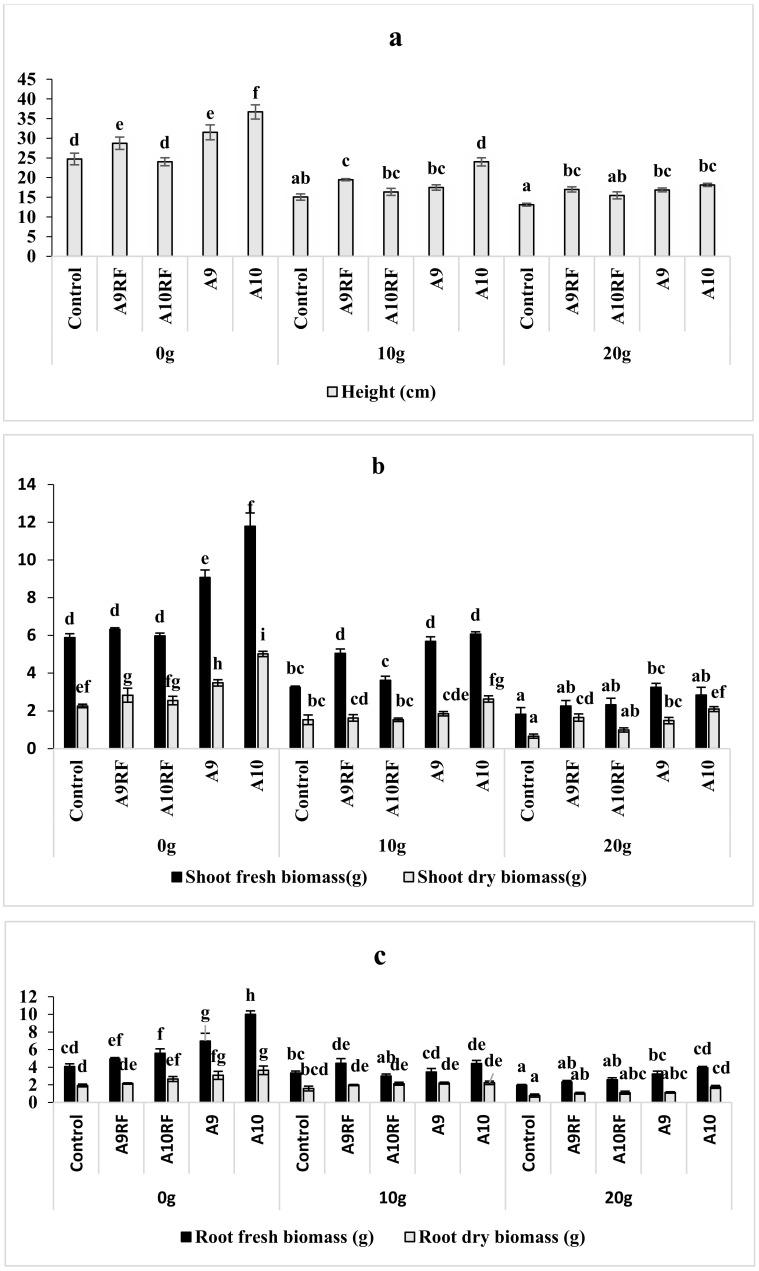
Effects of different fungal complexes and monospecific isolates on growth parameters [height (**a**), shoot biomass (**b**), root biomass (**c**)] of ten-month aged date palm seedlings under different salt stress levels. Graphs indexed by the same letter did not significantly differ according to Tukey (HSD) test (*p* < 0.05).

The application of salt stress showed a negative effect on the different morphological parameters. All the used inocula significantly ameliorate the growth of the date palm seedlings when compared to the non-inoculated ones (control) under different salt concentrations (Figure 2).

The application of salt stress (10 g·L^−1^ and 20 g·L^−1^ Na Cl) significantly, decreased plant height (*p* < 0.001). Therfore, the use of different fungal inocula improved the aerial elongation of plants in the cases of absence and presence of salt stress (Figure 2a). Fresh and dry shoot biomass of date palm seedlings that grew under salt stress was significantly (*p* < 0.001) lower when compared to those of unstressed plants. The supply of fungal inoculants to date palm seedlings inducted fresh and dry weight increase, which estimated to 60% for 0 g·L^−1^, 45% for 10 g·L^−1^, and 39% in the case of 20 g·L^−1^ (Figure 2b). At the root level, salinity negatively affected root fresh weight except for the non-stressed plants after ten months of cultivation. The application of AMF inoculants in the presence of salt stress showed significant improvement in root fresh and dry weights. Weighing of the root biomasses of inoculated and un-inoculated seedlings showed that the mycorrhizal complexes induced consistent improvements when compared to those of monospecific isolates under saline stress conditions (Figure 2c). In this sense, the mycorrhizal complex A9 isolated from the saline source showed the best growth performance.

### 3.3. Nutrient Contents

Phosphorus and nitrogen contents in date palm seedlings significantly decreased (*p* < 0.001) with the application of the salinity constraint. Thus, the greatest decreases in P and N contents were recorded in seedlings under 20 g·L^−1^ NaCl (Table 2). The concentrations of these elements were improved in the inoculated plants despite the application of salt stress and their concentrations were increased two times greater after ten months of cultivation when compared to the control. Na uptake was increased twofold in salt-stressed seedlings when compared to unstressed ones. Whereas, in the presence of fungal inoculants, a significant decrease in Na uptake was recorded. As expected, K and Mg uptake decreased by 44.57% and 31.11% at 10 g·L^−1^ and by 51% and 63% for 20 g·L^−1^ respectively (Table 2). Mycorrhizal seedlings showed higher values for K, Ca, and Mg even at higher salt concentration 20 g·L^−1^ compared to non-mycorrhizal seedlings.

### 3.4. Chlorophyll Contents

**Figure 3:**
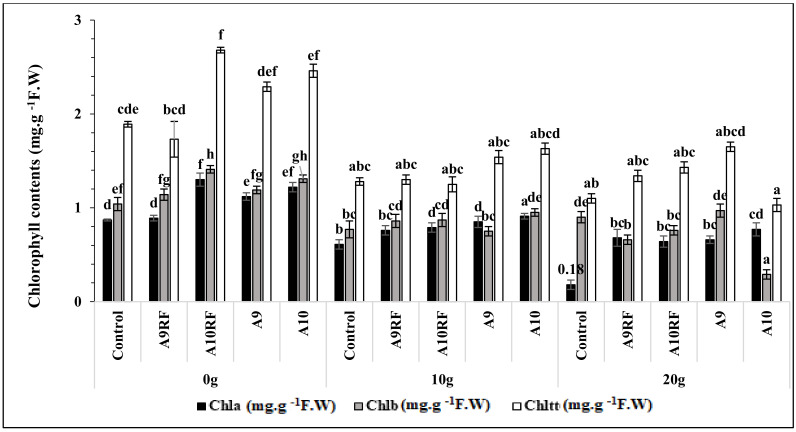
Effects of different fungal complexes and monospecific isolates on the chlorophyll contents of ten-month aged date palm seedlings under different salt stress levels. Graphs indexed by the same letter did not significantly differ according to Tukey (HSD) test (*p* < 0.05).

The data presented in Figure 3 show that the concentrations of the photosynthetic pigments decreased significantly (*p* < 0.001) due to salt stress. For instance, when plants were subjected to 20 g·L^−1^ NaCl, chlorophyll contents decreased strongly by 58% for Chl a, 51% for Chl b, and by 56%, for the total chlorophyll when compared to seedlings non exposed to salinity. However, the application of AMF positively counterbalanced the negative effects of salt stress and induced stimulating effects on the concentrations of the photosynthetic pigments when compared to non-mycorrhized seedlings. The largest increases in photosynthetic pigment contents among the salt-stressed seedlings were recorded when plants inoculated with the complex A9 at 20 g·L^−1^ NaCl, followed by the monospecific isolates A10RF, A9RF, and finally by the mixture A10. Faced with the same salt regime, mycorrhizal plants revealed a significant rise in these parameters after ten months of culture.

### 3.5. Malondialdehyde and Protein Contents

The interaction between AMF inoculation and salinity exhibited significant effects on protein and MDA levels in seedlings (*p* < 0.01) (Table 3). The results of salt stress effects on Malondialdehyde (MDA) and protein content of date palm seedlings are displayed in Table 3. From this table, it can be seen that the salinity significantly increased the MDA content in the seedlings when compared with the control sample (*p* < 0.001). In this sense, the largest increase (29%) was recorded for the non-inoculated stressed seedlings under 20 g·L^−1^. However, salt stress significantly decreased protein content in all the examined seedlings when compared with the control (*p* < 0.001). Inoculation of date palm seedlings with mycorrhizal complexes and monospecific isolates showed a significant decrease in MDA contents, so the best results were recorded with the complexes A9 and A10. Protein contents showed an increase of more than 23.5% in inoculated seedlings when compared with the control plants, especially in mycorhizal seedlings under 10 g·L^−1^ stress (Table 3).

### 3.6. Antioxidant Enzyme Activities

Salinity importantly increased the activities of the investigated antioxidant enzymes (Table 3). In this sense, it was registered that the activities of these enzymes were higher in seedlings especially under 10 and 20 g·L^−1^ NaCl stress. The inoculation of date palm seedlings with the different AMF inocula significantly increased SOD, PPO, and CAT bioactivities when compared with non-inoculated plants (*p* < 0.001) (Table 3). The best results were recorded with the selected complex A9 from the saline soil followed by the complex A10, isolated from date palm rhizosphere soil from Zagora region, and by the respective monopolar isolates A9RF and A10RF. The interaction between AMF inoculation and salinity exhibited a significant effect on the levels of antioxidant enzymes in the date palm seedlings so that the effect of AMF in salt stress tolerance was more appreciated (Table 3).

## 4. Discussion

The prospective role of AMF in the improvement of the tolerance of date palm to salt stress was examined in earlier work [22]. These scientific reports were conducted to evaluate and compare the effects of selected fungal inocula on morphological, physiological, and biochemical parameters of the date palm seedlings under salt stress. The obtained results showed that colonization by AMF of date palm seedlings was reduced by salt stress, especially when the applied concentration reached 20 g·L^−1^ NaCl. These results were in agreement with those reported elsewhere [41], which mentioned that increasing salt stress harms different mycorrhizal fungi colonization parameters of roots. However, the inoculation of seedlings with mycorrhizal fungi isolated from the saline soil improved the tolerance of seedlings to the salt constraint according to our findings. Therefore, our findings are in accordance with those reported in earlier work [42].

Previous studies have suggested that salinity induces a considerable reduction in date palm seedlings’ biomass [11,43]. In addition, the application of different inocula ameliorates the studied growth parameters, such as plant height, fresh and dry weight of shoots, and roots [43]. Generally, in the present work, the best results were obtained when the seedlings inoculated with the exotic complex A9 originated from a saline soil out of the date palm ecosystem and with the native complex A10 originated from the date palm ecosystem in the presence or absence of salt stress. In this sense, many works have shown that the AMF improves plant growth along with tolerance to salt stress [17,44]. It is thus fitting that, in this study, the increase in plant biomass can be attributed to the ability of AMF to enhance plant growth by developing external hyphae, which in turn increases soil surface area available for exploitation [42]. Moreover, several scientists have highlighted the beneficial effects of AMF on plant responses to salt stress [43,44,45].

In addition, AMF symbiosis and salinization did not affect the growth of date palm only, but also the mineral nutrient composition [46]. In this study, NaCl treatments caused a significant increase in Na^+^ contents, whereas P, K^+^, Mg^2+^, and Ca^2+^ content decreased significantly in non-mycorrhized date palm seedlings. Salinity inhibited the uptake of essential mineral elements including K^+^, Mg^2+^, and Ca^2+^ due to the antagonistic relationship modulated by the sodium [47]. Inoculation with AMF has been shown to better serve plant physiological processes, particularly nutrient uptake, and, therefore, plant tolerance to salt stress [48]. In this study, the amelioration of nutrient uptake (P, N, K, Mg, and Ca) under saline conditions was greater when seedlings associated with AMF inocula and particularly with the A9 complex were isolated from the saline soil followed by the native complex A10 and the respective monopolar isolates [43]. AMF have been reported to improve the N and P contents of plants grown in saline soils [49] and reduce the uptake of Na [50].

The current study showed that salinity reduced physiological parameters such as chlorophyll pigment contents. The salinity application significantly reduced the seedling’s contents of total chlorophylls, chlorophylls a and b. This reduction could be linked to chlorophyll-dehydration and chlorophyll-degrading enzymes activities such as chlorophyllases, which could be affected by a high level of salinity in the soil. AMF application improved chlorophyll pigment contents under saline and non-saline conditions. For comparison purposes, many studies have shown that AMF possesses a strong ability to decrease the salt stress effects on plant growth by improving the seedling’s photosynthesis activity [51,52]. Similarly, AMF inoculation augments plant tolerance to salt stress via enhancing antioxidant defense and chlorophyll content synthesis [53]. Indeed, AMF hyphae increase the uptake of nutrients such as Mg, which in turn augment the total chlorophyll content in mycorrhizal seedlings [53,54]. Additionally, MDA concentrations decreased, whilst the protein concentrations were raised in plants under salt stress conditions when compared with control plants [12,55,56]. In the present work, the best results were recorded when the seedlings were inoculated with the complexes A9 and A10. The significant augmentation in the antioxidant enzyme activity of the treated plants in the presence of salt stress probably resulted in a significant rise in MDA (stress markers) [57]. This decrease was more important in plants inoculated with the A9 complex [58]. It was reported that plants can tolerate salt stress through modification of biochemical responses: the improvement of protein biosynthesis and the decrease of MDA concentrations. It has also been observed that the use of AMF improves the quality of plants by improving their biochemistry through increasing the protein contents [58,59].

**Figure 4:**
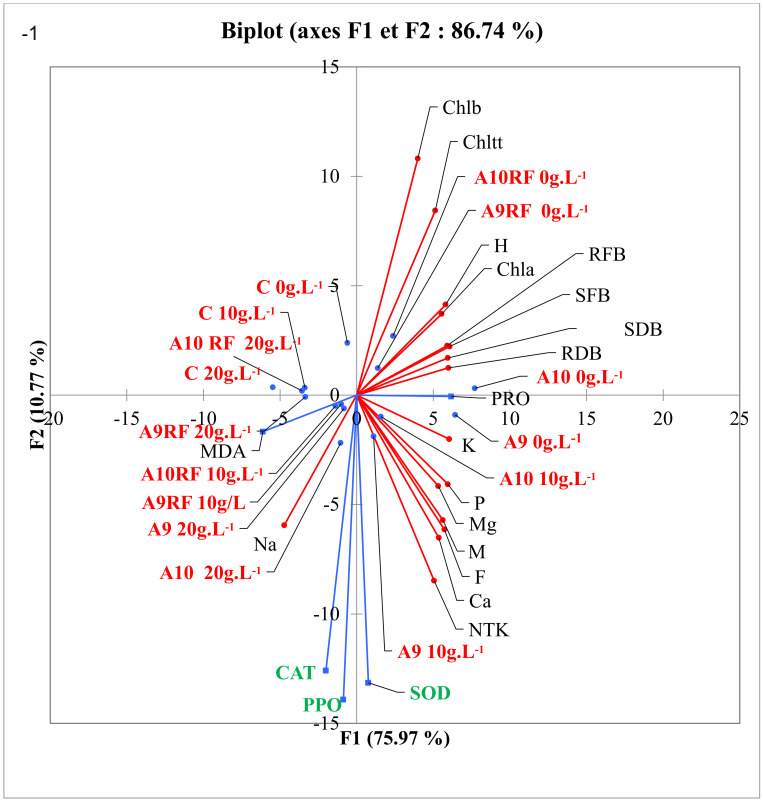
Graphical representation of the principal component analysis (PCA) of the correlation between the effects of AMF inoculants on growth, nutrition, protein, and MDA synthesis and oxidative enzyme activity of ten-month aged date palm seedlings under salt stress. A1, A4, A7, A9, A10 are arbuscular mycorrhizal fungi complexes; A1RF, A1RC, A4RF, A7RN, A9RF, A10RF are arbuscular mycorrhizal fungi monospecific isolates. H: Height; SFW: Shoot fresh biomass; SDB: Shoot dry biomass; RFB: Root fresh biomass; RDB: Root dry biomass; PRO: Protein contents; C: Control without arbuscular mycorrhizal inoculation.

In the present work, SOD, CAT, and PPO activities were raised under salt stress and increased more in the presence of AMF to reach higher levels. The Principal component analysis (PCA) showed that the grouping of fungal inocula on the one hand and of the measured variables on the other hand expressed strong correlations (F1: 75.97%) (Figure 4). These correlations were more marked especially between the fungal inocula A9 at 10 g l^−1^, A9RF and A10RF at 20 g·L^−1^, and the stress enzymes [12,14,60]. Indeed, the selected AMF could promote the activities of antioxidant enzymes as a protective strategy [12,57,61]. It is well recorded that salt stress favors the synthesis of ROS involved in oxidative stress [12]. Plants possess many defense mechanisms to fight against ROS. To achieve this goal, SOD is the primary defense enzyme that catalyzes the conversion of O to H_2_O_2_ and O_2_ [62]. The obtained findings showed a significant increase in SOD, CAT, and PPO in salt-stressed date palm seedlings inoculated with AMF when compared with control plants [12,57,61].

Many studies have shown that AMF has a great potential to enhance salinity tolerance by ameliorating both plasticity and stability of the plant cell membrane and activating the antioxidant defense mechanisms [58]. The application of AMF could reduce RNA disassembly and improve the capacity of the non-enzymatic antioxidant defense system by using soluble proteins [63].

The symbiotic interactions between the date palm tree and mycorrhizal fungi mixture in the saline soil led to the selection of performant fungal strains that could withstand the harsh conditions in this environment (Figure 4). Indeed, the establishment of such beneficial symbiotes in plant roots creates a new ecological niche previously defined as mycorrhizosphere. In this case, trophic interactions are modulated by the mycorrhizal fungi hyphae along with enzymatic activity of the fungal secretion involved in the biogeochemical cycles of the major nutrients in soil [64].

Previous works have highlighted the effects of mycorrhizal fungi complexes when compared to monospecific isolates. These results are linked to the genetic diversity in the complexes, and consequently to the combined effect of diverse fungal species coexisting in the mixture. The monospecific isolate A9RF extracted from saline soil showed higher propagation potential and noted effects on the tolerance of the date palm seedlings to saline stress.

Generally, the AMF communities showed higher potential for adaptation to different environmental conditions. However, when the abiotic and biotic constraints become extremely harsh, few fungal strains can outstand the pressure and then become endemic resources. Hence, their significant use as biofertilizers to promote soil fertility and plants growth in affected soils by anthropogenic factors and global changes is more appreciated [65].

## 5. Conclusions

In the present study, it was well demonstrated that the selected AMF contributed to the alleviation of salt stress in date palm seedlings. This could be explained by the active functioning of the mutualistic interaction between the fungi and this host plant. Mainly the hyphae of extra-radical fungi played a crucial role in water and mineral nutrition. In addition, mycorrhizal symbiosis promoted the increase of physiological and biochemical parameters as well as the production of antioxidant enzymes. These responses to salt stress are likely linked to the key beneficial role of AMF symbionts. This research argued that the date palm is a highly mycorrhizal-dependent species. Therefore, the use of native or exotic mycorrhizal complexes would be an effective ecological engineering method to treat date palm seedlings at an early stage in nurseries before transplantation to areas affected by salinity and climate change.

## Figures and Tables

**Table 1:** Geographical, physicochemical, and fungal spore richness of sampled rhizosphere soils of the date palm tree.

Sites	Soil Origin	Location	pH	Conductivity (ms·cm^−1^)	TOC (%)	TNK (%)	P (mg·kg^−1^)	Total Number of Spores 100 g^−1^ Soil
Saline site Marrakesh (A9)	Date palm tree	31.57 ± 7.67	7.05 ± 0.05	118.03 ± 1.97	3.67 ± 0.43	0.46 ± 0.14	12.24 ± 1.11	425 ± 70
Palm grove Zagora (A10)	Date palm tree	30.38 ± 6.21	8.23 ± 0.12	27.75 ± 0.22	0.96 ± 0.01	0.32 ± 0.21	4.45 ± 0.89	520 ± 15

**Table 2:** Effects of different fungal complexes and single-species isolates on the nutrient contents of ten-month aged date palm seedlings under different salt stress levels. The values represented by the same letter did not significantly differ according to Tukey’s test (HSD) (*p* < 0.05).

NaCl Treatment	Inoculum	P (mg·g^−1^)	NTK (%)	k (%)	Mg (%)	Ca (%)	Na (%)
0 g·L^−1^	Control	1.9 ± 0.89 ^ab^	0.66 ± 0.07 ^ab^	2.06 ± 0.07 ^c^	2.12 ± 0.18 ^d^	2.67 ± 0.19 ^bc^	2.14 ± 0.14 ^a^
	A9RF	4.27 ± 0.14 ^c^	0.91 ± 0.04 ^c^	2.79 ± 0.09 ^fg^	2.56 ± 0.1 ^e^	3.04 ± 0.56 ^cd^	2.43 ± 0.1 ^a^
	A10RF	5.79 ± 0.12 ^d^	0.8 ± 0.04 ^bc^	3.08 ± 0.05 ^gh^	1.35 ± 0.1 ^b^	3.98 ± 0.11 ^ef^	2.29 ± 0.13 ^a^
	A9	9.68 ± 0.24 ^f^	2.63 ± 0.07 ^f^	5.28 ± 0.05 ^j^	3.57 ± 0.09 ^g^	5.34 ± 0.07 ^h^	2.42 ± 0.15 ^a^
	A10	9.83 ± 0.11 ^f^	2.63 ± 0.1 ^f^	4.23 ± 0.09 ^i^	3.12 ± 0.05 ^f^	4.85 ± 0.08 ^gh^	2.56 ± 0.01 ^ab^
10 g·L^−1^	Control	1.51 ± 0.34 ^a^	0.58 ± 0.12 ^ab^	1.62 ± 0.1 ^b^	1.83 ± 0.09 ^c^	2.13 ± 0.09 ^ab^	5 ± 0.21 ^ef^
	A9RF	2.73 ± 0.29 ^b^	0.75 ± 0.07 ^bc^	2.62 ± 0.15 ^ef^	1.91 ± 0.06 ^cd^	3.92 ± 0.03 ^e^	4.73 ± 0.58 ^def^
	A10RF	2.55 ± 0.27 ^b^	0.66 ± 0.07 ^abc^	2.58 ± 0.1 ^ef^	2.11 ± 0.11 ^cd^	4.04 ± 0.08 ^ef^	4.38 ± 0.01 ^de^
	A9	7.43 ± 0.23 ^e^	1.98 ± 0.09 ^de^	2.79 ± 0.09 ^fg^	2.56 ± 0.1 ^e^	4.5 ± 0 ^fg^	3.43 ± 0.1 ^c^
	A10	6.81 ± 0.27 ^e^	2.11 ± 0.07 ^e^	3.08 ± 0.06 ^h^	2.17 ± 0.05 ^d^	4.18 ± 0.09 ^ef^	4.05 ± 0.66 ^cd^
20 g·L^−1^	Control	0.94 ± 0.17 ^a^	0.44 ± 0.05 ^a^	0.96 ± 0.13 ^a^	0.72 ± 0.09 ^a^	1.67 ± 0.32 ^a^	5.21 ± 0.01 ^f^
	A9RF	1.77 ± 0.38 ^ab^	0.58 ± 0.09 ^ab^	1.12 ± 0.11 ^a^	0.87 ± 0.1 ^a^	2.86 ± 0.07 ^cd^	4.38 ± 0.06 ^de^
	A10RF	1.4 ± 0.15 ^a^	0.56 ± 0.09 ^ab^	1.17 ± 0.06 ^a^	0.82 ± 0.04 ^a^	2.58 ± 0.09 ^bc^	4.44 ± 0.22 ^de^
	A9	4.47 ± 0.36 ^c^	1.86 ± 0.12 ^de^	2.38 ± 0.15 ^de^	1.89 ± 0.06 ^cd^	3.35 ± 0.09 ^d^	3.29 ± 0.16 ^bc^
	A10	3.96 ± 0.21 ^c^	1.75 ± 0.14 ^d^	2.22 ± 0.06 ^cd^	2.05 ± 0.1 ^cd^	3.93 ± 0.06 ^e^	3.48 ± 0.01 ^c^

*p* value (Two way ANOVA): Na Cl treatment *p* < 0.0001; Inoculum *p* < 0.0001; Na Cl Treatment Inoculum *p* < 0.0001.

**Table 3:** Influence of different salinity levels (0 g·L^−1^, 10 g·L^−1^, and 20 g·L^−1^) and different mycorrhizal inocula on MDA, protein, and oxidative enzyme activity (SOD), (CAT), (PPO) in non-inoculated (control) and inoculated ten-month aged seedlings. The values represented by the same letter did not significantly differ according to Tukey’s test (HSD) (*p* < 0.05).

Na Cl Treatment	Inoculum	MDA Level in (μM·g^−1^ of FM)	Protein (mg·g^−1^ of FM)	PPO (μmol de Catechol. min^−1^·mg^−1^ Prot)	SOD Activity (U·mg^−1^ of Protein)	CAT (U·mg^−1^ of Protein)
0 g·L^−1^	Control	26.07 ± 0.67 ^c^	11.7 ± 0.7 ^f^	0.64 ± 0.09 ^a^	1.14 ± 0.11 ^a^	1.47 ± 0.25 ^a^
	A9RF	24.09 ± 0.16 ^b^	14.12 ± 0.59 ^g^	0.94 ± 0.06 ^ab^	1.52 ± 0.45 ^a^	1.78 ± 0.11 ^ab^
	A10RF	23.53 ± 1.05 ^b^	13.95 ± 0.15 ^g^	0.76 ± 0.08 ^a^	1.38 ± 0.3 ^a^	1.66 ± 0.18 ^ab^
	A9	19.37 ± 0.44 ^a^	17.85 ± 0.71 ^h^	1.45 ± 0.09 ^def^	2.54 ± 0.27 ^c^	2.72 ± 0.15 ^ced^
	A10	18.35 ± 0.13 ^a^	16.97 ± 0.34 ^h^	1.41 ± 0.06 ^cde^	2.62 ± 0.08 ^c^	2.41 ± 0.1 ^bc^
10 g·L^−1^	Control	31.99 ± 0.66 ^g^	9.18 ± 0.34 ^bcd^	1.11 ± 0.09 ^bc^	1.58 ± 0.07 ^ab^	2.35 ± 0.49 ^abc^
	A9RF	30.19 ± 0.47 ^fg^	9.93 ± 0.21 ^de^	1.35 ± 0.12 ^cd^	1.78 ± 0.13 ^ab^	2.68 ± 0.27 ^cd^
	A10RF	29.54 ± 0.51 ^cd^	9.45 ± 0.62 ^cd^	1.39 ± 0.15 ^cde^	1.6 ± 0.18 ^ab^	2.47 ± 0.28 ^bc^
	A9	26.15 ± 0.71 ^c^	13.43 ± 0.62 ^g^	1.74 ± 0.1 ^f^	3.54 ± 0.19 ^d^	3.61 ± 0.35 ^ef^
	A10	26.68 ± 0.32 ^cd^	13.66 ± 0.36 ^g^	1.75 ± 0.12 ^f^	3.34 ± 0.3 ^d^	3.39 ± 0.34 ^def^
20 g·L^−1^	Control	37.28 ± 0.76 ^i^	6.56 ± 0.42 ^a^	1.45 ± 0.12 d^ef^	2.19 ± 0.11 ^bc^	3.46 ± 0.3 ^def^
	A9RF	35.68 ± 0.41 ^hi^	8.38 ± 0.36 ^bc^	1.73 ± 0.1 ^f^	2.48 ± 0.23 ^c^	3.65 ± 0.37 ^f^
	A10RF	34.76 ± 0.51 ^h^	8.01 ± 0.16 ^b^	1.68 ± 0.1 ^ef^	2.46 ± 0.09 ^c^	3.51 ± 0.21 ^def^
	A9	28.34 ± 0.78 ^de^	11.88 ± 0.16 ^f^	2.38 ± 0.1 ^g^	3.86 ± 0.1 ^d^	4.62 ± 0.47 ^g^
	A10	28.88 ± 0.88 ^ef^	11.14 ± 0.63 ^ef^	2.28 ± 0.13 ^g^	3.47 ± 0.2 ^d^	3.98 ± 0.24 ^fg^

*p* value (two-way ANOVA): Na Cl treatment *p* < 0.0001; Inoculum *p* < 0.0001; Na Cl treatment inoculum *p* < 0.0001.

## Data Availability

All data presented here are available from the authors upon request.
